# Selection of an optimal method for screening the collection of narrow-leaved lupine held by the Vavilov Institute for the qualitative and quantitative composition of seed alkaloids

**DOI:** 10.18699/VJ20.680

**Published:** 2020-12

**Authors:** A.V. Kushnareva, T.V. Shelengа, I.N. Perchuk, G.P. Egorova, L.L. Malyshev, Yu.A. Kerv, A.L. Shavarda, M.A. Vishnyakova

**Affiliations:** Federal Research Center the N.I. Vavilov All-Russian Institute of Plant Genetic Resources (VIR), St. Petersburg, Russia; Federal Research Center the N.I. Vavilov All-Russian Institute of Plant Genetic Resources (VIR), St. Petersburg, Russia; Federal Research Center the N.I. Vavilov All-Russian Institute of Plant Genetic Resources (VIR), St. Petersburg, Russia; Federal Research Center the N.I. Vavilov All-Russian Institute of Plant Genetic Resources (VIR), St. Petersburg, Russia; Federal Research Center the N.I. Vavilov All-Russian Institute of Plant Genetic Resources (VIR), St. Petersburg, Russia; Federal Research Center the N.I. Vavilov All-Russian Institute of Plant Genetic Resources (VIR), St. Petersburg, Russia; Federal Research Center the N.I. Vavilov All-Russian Institute of Plant Genetic Resources (VIR), St. Petersburg, Russia V.L. Komarov Botanical Institute of the Russian Academy of Sciences, St. Petersburg, Russia Saint-Petersburg State University, St. Petersburg, Russia; Federal Research Center the N.I. Vavilov All-Russian Institute of Plant Genetic Resources (VIR), St. Petersburg, Russia

**Keywords:** Lupinus angustifolius L., alkaloids, extraction techniques, lupanine, 13-hydroxylupanine, angustifoline, sparteine, isolupanine, Lupinus angustifolius L., алкалоиды, экстракция, люпанин, 13-гидроксилюпанин, ангустифолин, спартеин, изолюпанин

## Abstract

Narrow-leaved lupine (Lupinus аngustifolius L.) is a widely cultivated leguminous forage and green manure crop with a potential for human nutrition. However, the presence of secondary metabolites – alkaloids – in lupine seeds considerably affects the quality of raw produce, reducing its nutritive value; in addition, high concentrations of alkaloids are toxic to humans and animals. Therefore, plant breeders working with lupine need to gain knowledge about the variability of alkaloid content in seeds of different genotypes and search for the sources of their low concentrations in the crop’s gene pool. The collection of narrow-leaved lupine genetic resources held by the N.I. Vavilov Institute of Plant Genetic Resources (VIR) offers wide opportunities for such search by means of mass screening. For its part, largescale gene pool screening requires the selection of an optimal technique to measure alkaloid content in seeds, so that it would be easily reproducible and as little labor-, time- and fund-consuming as possible. The results of the search for such method are presented. Qualitative and quantitative indices were compared when target compounds had been extracted with multicomponent mixtures and individual reagents (chloroform, methanol, etc.) and the extracts analyzed using gas chromatography-mass spectrometry. High-performance liquid chromatography combined with mass spectrometry was also employed. Five major alkaloids were found to be present in all types of extracts: lupanine, 13-hydroxylupanine (dominant ones), angustifoline, sparteine, and isolupanine. The fullest extraction of alkaloids was observed when the extractant with an added alkaline agent was used (425 mg/100 g). The lowest level of extraction was registered with chloroform (216 mg/100 g). The significance of the differences was confirmed statistically

## Abstract (ru)

Люпин узколистный (Lupinus аngustifolius L.) – широко возделываемая кормовая и сидеральная зернобобовая культура с продовольственным потенциалом. Однако наличие в семенах люпина вторичных метаболитов – алкалоидов – значительно влияет на качество получаемого сырья, снижая его питательную ценность. Кроме того, высокие концентрации алкалоидов токсичны для человека и животных. Поэтому для селекции люпина актуальны сведения об изменчивости содержания алкалоидов в семенах у разных генотипов и поиск в генофонде источников их низкой концентрации. Коллекция генетических ресурсов люпина узколистного Всероссийского института генетических ресурсов растений им. Н.И. Вавилова предоставляет широкие возможности для такого поиска на основе массового скрининга. В свою очередь масштабный скрининг генофонда предполагает выбор оптимального метода оценки содержания алкалоидов в семенах: легко воспроизводимого, наиболее доступного с финансовой точки зрения и наименее трудо- и времязатратного. Представлены результаты поиска такого метода. Сравнивали качественные и количественные показатели при извлечении целевых веществ многокомпонентными смесями и отдельными реагентами (хлороформом, метанолом) и анализе экстрактов посредством газовой хроматографии, сопряженной с масс-спектрометрией. Также использовали высокоэффективную жидкостную хроматографию с масс-спектрометрией. Обнаружены пять алкалоидов, присутствовавших в экстрактах всех типов: люпанин и 13-гидроксилюпанин (доминирующие), ангустифолин, спартеин и изолюпанин. Наиболее полное извлечение алкалоидов отмечено при использовании экстрагента с добавлением щелочного агента – 425 мг/100 г. Минимальная экстракция зарегистрирована при извлечении хлороформом – 216 мг/100 г. Достоверность отличий подтверждена статистически.

## Introduction

Narrow-leaved lupine (Lupinus angustifolius L.) is a highprotein pulse crop, well adapted to comparatively low temperatures, acidic and meager soils. Its gene pool contains plenty early-ripening forms that reach maturity under a sum of active temperatures of 1700 °С, so its effective cultivation may be expanded practically to all regions of the Russian Federation (Artyukhov, 2015; Ageeva et al., 2018). It is chiefly used as a fodder and green manure crop, but there are prospects of its utilization for food production (Krasilnikov, Pankina, 2006; Islam et al., 2011). The cost price of lupine grain production is twice lower than that of soybean (Korol’, Lahmotkina, 2018). However, the production of feed and food from most of the existing genetic resources of Lupinus angustifolius L. is restricted by the presence of secondary metabolites – alkaloids – in their seed and biomass. The collection of narrow-leaved lupine maintained at N.I. Vavilov All-Russian Institute of Plant Genetic Resources (VIR) was found to harbor a considerable variability of alkaloid content levels in its seeds (Kurlovich et al., 1995). For many years, since the 1960s, such assessment had been performed on an overwhelming majority of accessions employing the rapid method of in-field differentiation between high-alkaloid and low-alkaloid varieties with Dragendorff’s reagent (Ermakova et al., 1987). For part of the accessions, the data were retrieved from published sources and descriptions of varieties submitted by plant breeders. It means that exact quantitative characterization of alkaloid content is absent for most of the accessions preserved in the collection.

Alkaloids, being biologically active compounds, have a negative effect on human and livestock organisms, worsen organoleptic properties of lupine-based products, and reduce the value of its seeds as raw materials for food and feed (Cheeke, Kelly, 1989; Resta et al., 2008). According to the production standards accepted in Russia, the content of alkaloids in lupine seeds earmarked for food and feed purposes should not exceed 0.04 % (40 mg/100 g) of the seed weight (Kuptsov, Takunov, 2006); in some European countries and Australia, no more than 0.02 % (20 mg/100 g) (Frick et al., 2017). This gave a stimulus to one of the prioritized trends in lupine breeding, aimed at the development of low-alkaloid cultivars.

Scientific plant breeding to improve this species, per se, started in the first third of the 20th century, after the development of the first low-alkaloid lupine cultivar Stamm 411 by the German researcher R. Sengbusch in 1928 (Sengbusch, 1931). By now, a substantial number of narrow-leaved lupine cultivars have been released, with the alkaloid content in their seeds not exceeding permissible levels (Kuptsov, Takunov, 2006).

It should be mentioned that lupine alkaloids are widely used in medicine and pharmacology as ganglionic blockers, antiarrhythmic agents, etc. (Hatzold et al., 1983). At present, the antimicrobial effect of lupine alkaloids is actively researched (Erdemoglu et al., 2007), so there is also a need to search for accessions with increased concentrations of total alkaloids or with higher contents of individual compounds within this group (Williams et al., 1984).

Up to 120 alkaloids have been identified in the composition of plant tissues in Lupinus angustifolius. Among them, lupanine is the dominating one (50–70 % of the total alkaloids); the shares of 13-hydroxylupanine and angustifoline are ca. 12–30 and 10 %, respectively (Frick et al., 2017). Minor lupine alkaloids, such as pachycarpine, lupinin and matrine, are chemical modifications of the above mentioned alkaloids (Wink, 1987). Qualitative composition of alkaloids does not differ across various forms and varieties of narrow-leaved lupine: all accessions contain the same alkaloids in different proportions (Krasilnikov, Pankina, 2006).

Selection of a method for extraction of these compounds from raw plant material is a paramount stage in the biochemical analysis of alkaloids, determining the accuracy of results during quantitative and qualitative evaluation. The ways to extract alkaloids rapidly from small amounts of material are quite numerous (Adejoke et al., 2019). However, each of them has its individual drawbacks: low performance, very laborconsuming operations, a need for large amounts of toxic and expensive solvents, etc. (Zharylgasina et al., 2014). Besides, most of these techniques would require the use of alcohols, ionic liquids or other solvents non-selective in their hydrophilic/hydrophobic pattern, which complicates purification of alkaloids by removing admixed low-molecular-weight metabolites (mono- and oligosaccharides, alcohols, free amino acids, organic acids, etc.).

Presently, the efficiency and intensity of extraction processes are increased using alternative, resource-saving technologies, specifically microwave/ultrasound treatment (Popova, Potoroko, 2018).

Basic methods to evaluate qualitative and quantitative alkaloid composition are high-performance liquid chromatography (HPLC) and gas chromatography coupled to mass spectrometry (GC-MS). Most researchers favor HPLC-MS. Gas chromatography, however, makes it possible to achieve the same resolution and precision with less time and labor expenditures. Liquid chromatography prefers extracts containing alkaloids in the form of salts, while gas chromatography in the form of bases (Markova et al., 2003).

This paper describes and characterized the alkaloid extraction techniques tested by us on narrow-leaved lupine seeds to select an optimal one for identification of their qualitative and quantitative composition by means of GC-MS, suitable for mass screening of accessions from the lupine collection held by VIR.

## Materials and methods

The research material were seeds of cultivar “Оligarkh”, a high-alkaloid green-manure variety of narrow-leaved lupine (k-3814) from the collection of the N.I. Vavilov Institute of Plant Genetic Resources (VIR), grown according to the guidelines developed by VIR (Vishnyakova et al., 2018), on the experimental fields in the town of Pushkin (St. Petersburg) in 2016, and harvested in the phase of full ripeness.

Four ways to obtain alkaloid-containing extracts were tested in the process of research (hereinafter: A, B, C and D procedures). Before extraction, lupine seeds were crushed with a hammermill into thinly dispersed flour. Then alkaloids were extracted from the flour by the following methods.

Extraction procedureA makes it possible to produce alkaloids in the form of bases (Mironenko, 1966). It was divided into two versions, A1 and A2:

А1 – 2 g of lupine seed flour was mixed with diethyl ether, supplemented with chloroform and a 5 % water solution of NaOH in ratio 10 : 5 : 1;

A2 – 2 g of flour was mixed with ethyl acetate, supplemented with a concentrated ammonia solution in ratio 8 : 1.

Both solutions, A1 and A2, were treated according to the same pattern: they were left for 16–18 hours at 4 °C; after that, they were filtered through Whatman ash-free filter paper (0.45 μm, Merck, Germany) and Millipore polytetrafluoroethylene syringe filters (diameter 25 mm, pore size 22 µm, Ireland) to remove solid residues of plant material. As a result, samples A1 and A2 were produced. 

Procedure В was employed to obtain acid salts of alkaloids: 10 mL of sample A1 was mixed with a 1 % water solution of hydrochloric acid in ratio 1 : 1. After that, the water layer containing alkaloid salts was isolated (sample B) (Mironenko, 1966).

The С and D techniques make it possible to extract native forms of alkaloids as salts of organic acids using certain extractants (methanol or chloroform).

Procedure С is an intensified technique to obtain native forms of alkaloids, based on ultrasound application. Lupine seed flour (0.3 g) was mixed with methanol (1 mL). The resulting mixture was treated for 30 min with ultrasound in an Elmasonic S30H bath (Germany), ultrasonic wave length 220 nm, and later infused for 8 hours at +4–6 °C (sample C).

Procedure D: lupine seed flour (250 mg) was mixed with chloroform (1 mL). The mixture was infused for 16–18 hours at +4–6 °C (sample D) (Zharylgasina et al., 2014).

After infusion, samples C and D were centrifuged for 15 min on an Eppendorf 5415C Centrifuge (Germany) at 8000 rpm. The supernatant was collected for further analysis. Then, 100 μl portions of samples A1, B, C and D were dried according to the same pattern using the vacuum concentrator Savant™ SpeedVac™ (USA).

The resulting solid residues of samples A1 , B, C and D were silylated by adding 20 μl of N,O-Bis(trimethylsilyl)trifluoroacetamide. The mixture of trimethylsilyl ethers was separated on an Agilent HP-5MS capillary column (5 % phenyl, 95 % methylpolysiloxane; 30.0 m, 250.00 μm, 0.25 μm), at the inert gas speed of 1.5 mL/min, employing a gas chromatograph (Agilent 6850 Network GC System) with a quadrupole mass-selective detector (Agilent 5975B VL MSD), produced by Agilent Technologies, Inc. (USA). Heating program: from +170 to +320 °C, heating rate: 4 °C/min. Mass spectrometer detector temperature: +250 °C, injector temperature: +300 °C, sample size: 1.2 μl. Sample A2 was analyzed with GC-MS without additional conversion (without silylation).

Acid salts of alkaloids (sample B) were separated using a liquid chromatograph (Agilent Technologies Series 1200, USA) on an Agilent Zorbax SB-C18 column (150 mm; 3 mm; 1.8 μm) at a gradient elution mode from 1.000 to 0.425 deionized water/acetonitrile. Elution speed: 50.00 μl/min. Sample size: 0.5 μl.

The following commercial standards were used to identify alkaloids: 900263 for sparteine (Sigma-Aldrich, USA); ALBRS-1465 for lupanine (ALB Technology Limited, USA); sc-481026 for angustifoline, and sc-490845 for 13-hydroxylupanine (Santa Cruz Biotechnology, USA). As an internal standard for quantitative calculation of alkaloid content, the commercial standard for caffeine, 142833 (PanReac AppliChem, ITW, USA) was used, in the 1 μg/μl concentration.

Statistical analysis. The results obtained were processed using the AMDIS and UniChrom software. Statistical data processing was made using the Statistica 7.0 software package; it included ANOVA and factor analysis of correlation matrix.

## Results

**Fig. 1.**
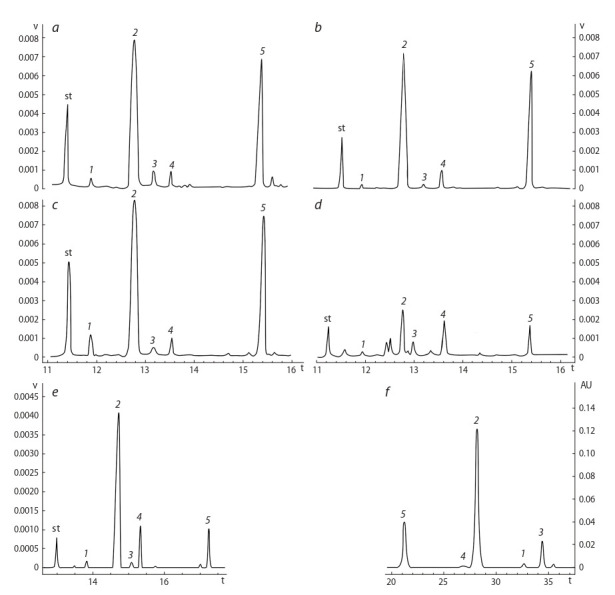
Chromatograms of the samples: a – А1; b – А2; c – C; d – D; e – B, obtained by GC-MS; f – chromatogram of acid salts of the alkaloids obtained by HPLC-MS. Alkaloids: st – standard; 1 – sparteine; 2 – lupanine; 3 – angustifoline; 4 – isolupanine; 5 – 13-hydroxylupanine.

Five alkaloids typical for lupine seeds were identified in the extracts produced by all tested extraction procedures (А1, А2, В, С and D) where GC-MS was used: lupanine, 13-hydroxylupanine, sparteine, angustifoline and isolupanine (Fig. 1, a–e). To control the fullness of alkaloid extraction from the plant material under different techniques of quantitative sample preparation with GC-MS, acid salts of alkaloids (sample B) were analyzed by means of HPLC, because this type of chromatography is most frequently used while studying plant alkaloids (see Fig. 1, f   ). In this case, the same set of alkaloids was identified as with GC-MS. The analysis of acid salts (sample B) with HPLC showed that the amounts of lupanine, 13-hydroxylupanine, angustifoline, sparteine and isolupanine were 259.63; 46.51; 56.00; 20.87 and 2.31 mg/100 g (67.38; 12.07; 14.53; 5.42 and 0.6 % of the total alkaloids), respectively. Using GC-MS to analyze sample B demonstrated practically the same results (257.93; 46.23; 54.92; 20.12 and 2.07 mg/100 g, or 67.65; 12.13; 14.40; 5.28 and 0.54 %, respectively).

Lupanine was the dominating alkaloid in all samples analyzed with GC-MS: its content varied from 317.86 (sampleA1) to 196.43 mg/100 g (sample D), or from 77.60 to 90.63 % of the sum of all alkaloids identified in the respective samples. The next in amount in the alkaloid composition of lupine seeds were 13-hydroxylupanine and angustifoline. The content of 13-hydroxylupanine varied from 9.67 (sample D) to 58.42 mg/100 g (sample A2), which corresponded to 4.46 and 13.72 %. The content of angustifoline ranged from 54.92 (sample B) to 9.33 mg/100 g (sample D), and from 14.40 to 4.30 % (sample D). The levels of sparteine were significantly lower: from 20.12 (sample B) to 1/10 mg/100 g (sample D), corresponding to 5.28 and 0.51 %, respectively. The minimum content was recorded for isolupanine: from 2.07 (sample B) to 0.12 mg/100 g (sample C), or 0.54 and 0.05 % (Table).

**Table 1.**
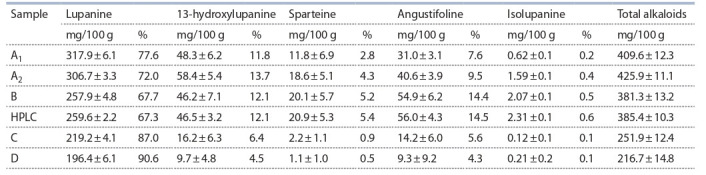
The content of main alkaloids in the seeds of the lupine cultivar “Оligarkh” under various extraction options, mg/100 g, and % of the total amount of identified alkaloids Note. HPLC – high-performance liquid chromatography.

The results confirmed that the qualitative and quantitative composition as well as the ratios of alkaloids in the samples practically coincided in both, GC-MS and HPLC, versions (see Fig. 1, c, f ). Thus, alkaloid extraction from lupine seeds had the best outcome with the A2 and B techniques, while the C and D extraction procedures mostly isolated the dominant alkaloid, lupanine.

The least labor- and time-consuming alkaloid extraction procedure for lupine seeds was the C technique (only nine hours, requiring direct involvement of a researcher). Time expenditures for the А1, А2, В and D procedures were almost the same (from 19 up to 22 hours).

The most laborious way of sample preparation (the largest number of manipulations) was producing acid salts of alkaloids (the B technique).

**Fig. 2.**
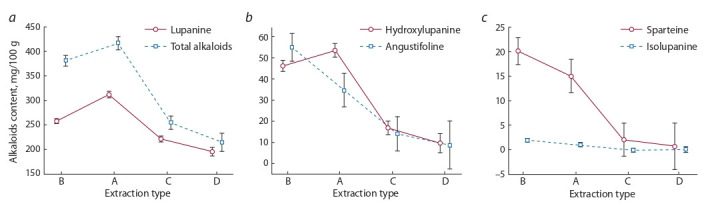
. ANOVA of the content (mg/100 g) of the total alkaloids and lupanine (a), 13-hydroxylupanine and angustifoline (b), sparteine and isolupanine (c) in the seeds of the lupine cultivar “Оligarkh” measured under various extraction techniques (A, B, C and D).

The analysis of variance showed that the alkaloid extraction techniques tested by us on narrow-leaved lupine seeds had significant differences among them, both in the total alkaloid content and in concentrations of individual compounds (Fig. 2). For example, theA procedure in both versions (А1 and А2) proved the most efficient for extracting dominating alkaloids, i. e., lupanine and 13-hydroxylupanine, and the sum of alkaloids (see Fig. 2, a). To isolate sparteine and angustifoline, the B technique was the best (see Fig. 2, c, b). The amount of isolupanine extracted with all sample preparation techniques did not exceed 2.2 mg/100 g and was the lowest. In the case of isolupanine content, no significant differences were observed among the tested techniques (see Fig. 2, c). The use of the C and D procedures demonstrated the lowest values of both the sum of extracted alkaloids and their individual fractions, so they proved to be the least effective (Fig. 2, a–c).

Analyzing the system of correlations between the content and the percentage of the identified alkaloids under different extraction techniques helped to identify two factors, embracing 95.9 % of the variation in the set of the data obtained.

**Fig. 3.**
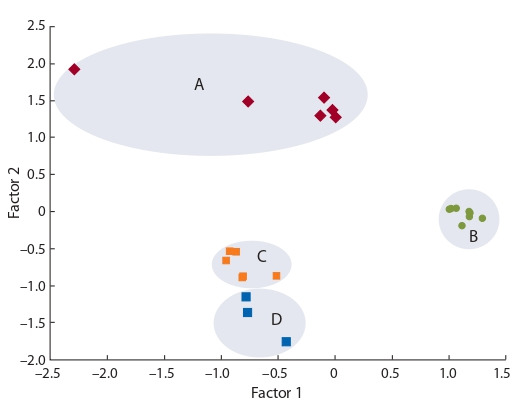
Results of a factor analysis of alkaloid composition and content in the seeds of the lupine cultivar “Оligarkh” measured under various extraction techniques (A, B, C and D).

Factor 1 (58.5 % of variability) was associated with the variations in the content and percentage of sparteine, angustifoline and isolupanine, while factor 2 (37.4 %) with that of lupanine and 13-hydroxylupanine. The A and B extraction techniques were found to differ considerably in the factor structure of the variables from the C and D ones. The C and D procedures formed a separate group, because the results obtained with them had no statistically significant differences between them (Fig. 3).

## Discussion

Lupine alkaloids are attributed to the quinolizidinic group and contain one (lupanine and 13-hydroxylupanine) or two (sparteine) condensed quinolizidine nuclei. For all lupine alkaloids, their molar mass does not exceed 300 g/mol. A peculiar structural feature of an alkaloid molecule is the presence of an undivided pair of electrons in a nitrogen atom (Orekhov, 1955; Roberts, Wink, 2013), which explains their properties that determine the specific nature of the techniques of their extraction from plant tissues. Alkaloids are present in plants mostly as salts, because they interact with organic acids contained in plant cells, which should be taken into account while selecting an extraction method (Mironenko, 1966). In the form of bases, alkaloids are readily soluble in chloroform, ether or ethyl acetate, but practically insoluble in water; on the contrary, in the form of salts, they are water-soluble, but insoluble in organic solvents. A water solution of NaOH or, less frequently, ammonia is used as an alkaline agent for extraction of alkaloids as bases. Ultrasound is used to improve the extraction kinetics and increase the outcome of the target product (Vilkhu et al., 2008; Popova, Potoroko, 2018).

Considering these specific features, we applied different extractants to retrieve alkaloids from narrow-leaved lupine seeds: both hydrophobic (chloroform, ethyl acetate, and diethyl ether) and hydrophilic ones (methanol, and water solution of hydrochloric acid). All alkaloids typical for the studied species were identified in all extracts produced under all versions of sample preparation (Krasilnikova, Pankina, 2006; Erdemoglu et al., 2007). With all applied extraction techniques, lupanine Considering these specific features, we applied different extractants to retrieve alkaloids from narrow-leaved lupine seeds: both hydrophobic (chloroform, ethyl acetate, and diethyl ether) and hydrophilic ones (methanol, and water solution of hydrochloric acid). All alkaloids typical for the studied species were identified in all extracts produced under all versions of sample preparation (Krasilnikova, Pankina, 2006; Erdemoglu et al., 2007). With all applied extraction techniques, lupanine

In our research, under all sample preparation techniques, lupanine was present in the extracts in maximum amounts – from 67.4 to 96.0 %. At the same time, the amount of sparteine was many times (from 20 to 200, or more) lower, depending on the way of extraction. Therefore, we assumed that the least expensive sample preparation procedures, when mostly lupanine was extracted, might be used for a screening assessment of “alkaloidization” in large numbers of accessions, i. e., a collection of narrow-leaved lupine genetic resources. Concentrations of lupanine in seeds, measured by such techniques, would help to understand whether such alkaloid content should be deemed fit to regard the lupine variety in question suitable for food or feed purposes. It was observed in our research that simplifying the composition of extractants to a single component, comparted with theA procedure where they were multicomponent, led to an almost twofold reduction in the extraction of the total alkaloids (see Fig. 2, a). The most laborious and lengthy technique, when alkaloids were extracted in the form of salts, was the B procedure. When applied, this technique led to lesser extraction of the dominating alkaloids in narrow-leaved lupine (lupanine and 13-hydroxylupanine) than with theA technique, but sparteine and angustifoline were isolated to a greater extent than with the other methods. The total sum of alkaloids was lower than with the extraction by theA technique, but higher than with the C and D procedures, which proved less labor-consuming and the most cost-effective as far as financial aspects are concerned.

## Conclusion

A comparison among all tested techniques of alkaloid extraction from narrow-leaved lupine seeds has shown that the A procedure (versions A1 and A2) seems the most effective for quantitative assessment. This technique, involving multicomponent extractants containing an alkaline agent, has sufficient capacity and good reproducibility, which gives enough reason to regard it as reliable for evaluation of germplasm collection holdings and for most precise measurement of total alkaloid concentrations and amounts of individual alkaloids. However, as an alternative way to perform mass screening of large numbers of accessions, it is possible to employ the sample preparation procedure where only one solvent is used (methanol or chloroform). It will enable a researcher to measure the amount of lupanine, the dominant alkaloid in narrow-leaved lupine seeds, identify a permissible alkaloid content determining food or feed purposes of an accession, and categorize accessions from the collection according to their alkaloid content (high-, medium- and low-alkaloid). Despite the fact that most researchers use HPLC-MS to identify alkaloids, our study has shown that gas chromatography may be used with the same resolution and accuracy, but requires less time and labor inputs

## Conflict of interest

The authors declare no conflict of interest.
